# Cross-Selectivity Enhancement of Poly(vinylidene fluoride-hexafluoropropylene)-Based Sensor Arrays for Detecting Acetone and Ethanol

**DOI:** 10.3390/s17030595

**Published:** 2017-03-15

**Authors:** Ali Daneshkhah, Sudhir Shrestha, Amanda Siegel, Kody Varahramyan, Mangilal Agarwal

**Affiliations:** 1 Department of Electrical and Computer Engineering, Indiana University–Purdue University Indianapolis (IUPUI), Indianapolis, IN 46202, USA; alidane@umail.iu.edu (A.D.); shrests2@miamioh.edu (S.S.); kvarahra@iupui.edu (K.V.); 2 Integrated Nanosystems Development Institute (INDI), Indiana University–Purdue University Indianapolis (IUPUI), Indianapolis, IN 46202, USA; apsiegel@iupui.edu; 3 Department of Mechanical Engineering, Indiana University–Purdue University Indianapolis (IUPUI), Indianapolis, IN 46202, USA

**Keywords:** acetone sensor, ethanol sensor, volatile organic compounds, PVDF-HFP/carbon black composite, low water sensitivity

## Abstract

Two methods for cross-selectivity enhancement of porous poly(vinylidene fluoride-hexafluoropropylene) (PVDF-HFP)/carbon black (CB) composite-based resistive sensors are provided. The sensors are tested with acetone and ethanol in the presence of humid air. Cross-selectivity is enhanced using two different methods to modify the basic response of the PVDF-HFP/CB sensing platform. In method I, the adsorption properties of PVDF-HFP/CB are altered by adding a polyethylene oxide (PEO) layer or by treating with infrared (IR). In method II, the effects of the interaction of acetone and ethanol are enhanced by adding diethylene carbonate (DEC) or PEO dispersed in DEC (PEO/DEC) to the film. The results suggest the approaches used in method I alter the composite ability to adsorb acetone and ethanol, while in method II, they alter the transduction characteristics of the composite. Using these approaches, sensor relative response to acetone was increased by 89% compared with the PVDF-HFP/CB untreated film, whereas sensor relative response to ethanol could be decreased by 57% or increased by 197%. Not only do these results demonstrate facile methods for increasing sensitivity of PVDF-HFP/CB film, used in parallel they demonstrate a roadmap for enhancing system cross-selectivity that can be applied to separate units on an array. Fabrication methods, experimental procedures and results are presented and discussed.

## 1. Introduction

Volatile Organic Compounds (VOCs) are present in the air in varying concentrations. As chronic exposure to some VOCs can cause ill effects to human health, it is important that reliable detection methods be developed and utilized. Cross-selective sensors can be applied to screen for VOCs in numerous applications. Resistive sensor arrays for detecting organic compounds are primarily developed on interdigitated electrodes (IDEs) of gold over glass, silicon oxide, and polymer substrates [1,2]. The adsorption of VOCs on the surface of sensing materials changes the resistance of the films. Sensor arrays consisting of functionalized carbon nanotubes (CNTs), graphene, conductive polymers, carbon black, metal oxide, gold nanoparticles, and conducting polymer composites have been studied extensively for their ability to detect organic compounds [1,2,3,4,5,6]. The variance in single wall carbon nanotube (SWNT) chiral distributions makes it difficult to fabricate sensors reproducibly [5]. CNT conductance is sensitive to temperature, humidity and air flow speed [6]. In addition, a relatively high response to oxygen, carbon dioxide and water vapor present in the environment can be major drawbacks for CNT- and graphene-based VOC sensors [7,8]. Response to water vapor and dependency on temperature are undesirable features of metal oxide and monolayer-capped gold nanoparticles [2,9,10,11,12]. Sensor degradation is a concern for monolayer-capped gold nanoparticle-based sensors, and sensors fabricated from conducting polymers such as polypyrrole (PPy) and polyaniline (PANI) [13,14,15]. Composites of polymers with conductive fillers offer good stability and have widely been used for the development of electronic noses [16,17,18]. These sensors comprise a polymer that senses the gas, and a conducting filler such as CB to increase conductivity. Swelling of the polymer due to interactions with VOCs increases the separation between the conducting particles which in turn increases the film resistivity [19,20]. A thick film can be developed over the IDE via a drop-casting method [16,17]. In order to change the cross selectivity of the sensors in an array of sensors, different combinations of polymers are used [16,18]. Reproducible development of multiple thin films on a single substrate is a challenging task. These challenges have drawn much attention and methods such as automatic thin film transferring are investigated [21,22]. One simple solution is to change the selectivity of the thin film after sensor development. PVDF-HFP is well known for its applications in piezoelectric devices and batteries. Its high stability and hydrophobicity with low degradation and good resistivity against many chemicals make it a good candidate for making selective resistor based gas sensors [2]. Fabrication of a thin film PVDF-HFP/CB results in the development of reliable sensors to detect VOCs while rejecting water vapor [2]. However, these sensors currently lack the cross-selectivity needed for them to be effective in a sensor array. Detecting VOCs with specificity will require an array of cross-selective sensors [2,16,18]. Here we present methods of enhancing the cross-selectivity of PVDF-HFP-based sensors seeded with CB in a single thin film. PVDF-HFP/CB composite can adopt three crystalline structures, α, β, and γ [23]. The different polymeric composite structures adsorb VOCs differently. Effectively, the β and γ structures are more polar, while the α one is more symmetrical and nonpolar [23]. In this paper, we report methods of enhancing cross-selectivity of porous PVDF-HFP/CB-based sensors by varying the surface and structural properties of the film.

One hallmark of cross-selectivity is how a sensor responds to different volatile organic compounds. For this study, we will demonstrate the results of cross-selective enhancements utilizing a polar compound, ethanol, and a nonpolar compound, acetone. Sensitivity of the PVDF-HFP/CB sensors described herein are similar to those reported in other studies as evidenced by examples shown in Table 1.

## 2. Materials and Methods 

CB (Black Pearl 2000) was obtained from Cabot Corporation (Boston, MA, USA). PVDF-HFP (FLEX 2801 PVDF), obtained from Arkema Group Kynar (Colombes, France) is comprised of PVDF with around 3.5%–7.5% HFP. Diethylene carbonate (DEC) was purchased from Novolyte Technologies (Zachary, LA, USA). 1-Methyl-2-pyrrolidinone (NMP) anhydrous, glycerol anhydrous and polyethylene oxide (PEO) (average Mv 4,000,000), were purchased from Sigma Aldrich (St. Louis, MO, USA). The composite of PVDF-HFP/CB/glycerol (20:1:4 *w*/*w*) is synthesized in NMP. The sensors were fabricated using IDEs of gold (25 µm spacing) over silicon oxide. A standard photolithography process was used to fabricate the IDEs [2]. Spin-coating method was used to cast a thin film of synthesized materials slurry over the IDEs. The film thickness is estimated to be less than 4 µm. Next, the film was dried slowly in ambient temperature followed by washing with water to remove the glycerol from the composite. The PVDF-HFP and CB form a conducting porous film. To better understand the contribution of CB in PVDF-HFP/CB sensors, CB only sensors were also fabricated via drop-casting of CB in NMP directly onto the IDE.

**Figure 1:**
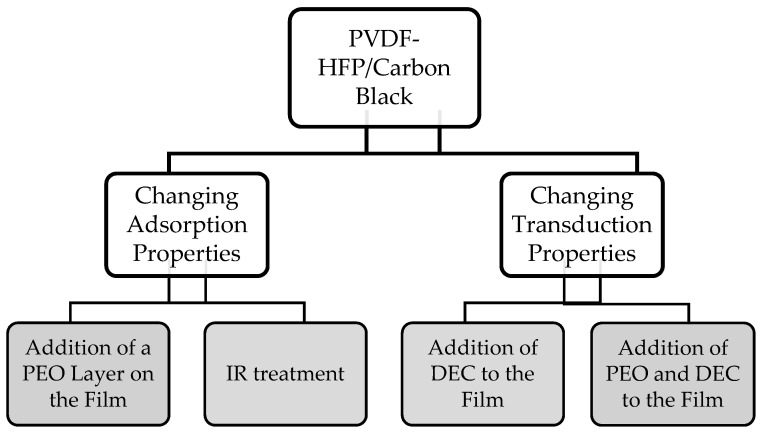
Presented methods to enhance cross selectivity of the porous thin PVDF-HFP/CB film.

PVDF-HFP swells due to interactions with VOCs, and this increases the separation between the conducting particles, thereby increasing film resistivity. There are two major factors that determine a sensor’s ability to detect a specific VOC. First, the ability of the sensing material to adsorb the VOC (defined as adsorption efficiency) and, second, the effects of interactions of adsorbed compounds with the composite in changing the film resistivity (defined as transduction efficiency). Enhancement in adsorption efficiency leads to more interaction between the VOC and the film which increases the swelling of the film and thus the film resistivity. Enhancement in transduction efficiency causes more resistance change with the adsorbed VOCs which increases the sensitivity. These factors have been utilized in the presented two methods (method I and method II) to enhance the cross-selectivity of PVDF-HFP/CB composite films. Method I seeks to alter adsorption properties (adsorption efficiency) and method II seeks to alter the transduction properties (transduction efficiency). In method I, a thin PEO layer was cast over the film, or the film was treated with an IR lamp. The addition of PEO layer enhances ethanol adsorption while an IR treatment reduces it. In method II, transduction efficiency was improved for both ethanol and acetone through the addition of DEC or PEO/DEC to the film. The addition of DEC increased the sensitivity to both acetone and ethanol, whereas the addition of PEO/DEC skewed it toward ethanol. These processes are further described in Figure 1, shown below.

### 2.1. Method I

The enhancement in the composite ability to adsorb VOCs causes more VOCs to interact with the composite, thereby increasing adsorption efficiency by increasing the resistance change in response to VOCs. Method I uses two approaches to alter the ability of the PVDF-HFP/CB film to adsorb and capture VOCs. These two approaches are addition of a PEO layer on the film and IR treatment. The two approaches and their potential to alter adsorption ability of the composite are unpacked. 

#### 2.1.1. Addition of a PEO Layer on the Film 

The slow evaporation of the film causes formation of microscale pores in the PVDF-HFP/CB composite. These pores can be filled with polymers to attract VOCs. In the presented work, a PEO layer was added to the composite film by drop-casting a solution of high molecular weight PEO. A water bath was then used to wash away the bulk of PEO from the composite and leave a very thin layer. A quartz crystal microbalance (QCM) experiment before and after the addition of PEO layer was used to confirm the formation of a thin PEO layer on the PVDF-HFP/CB. PEO strongly attracts VOCs such as ethanol and acetone [33,34,35]. Thus the fabricated thin PEO layer on the PVDF-HFP/CB composite adsorbs ethanol and acetone differently than PVDF-HFP/CB alone. 

#### 2.1.2. IR Treatment of the Film 

PVDF-HFP has a property whereby it can offer either a more polar crystalline structure (β-phase and γ-phase), or less polar crystal structure (α-phase) [2,23,36]. The IR treatment of PVDF-HFP in the presence of CB particles has been investigated. Sensors were exposed to a 200 W IR lamp at a distance of 10 cm for one hour immediately after the material was cast on them. A mask was used to protect half of the sensors from IR radiation. Treatment of PVDF-HFP/CB with IR alters the crystal structure of the film. The change in PVDF-HFP/CB crystalline structure and polarity alters the composite’s tendency to adsorb the more polar ethanol and less polar acetone. 

### 2.2. Method II

Enhancement in transduction efficiency increases sensor’s response to a fixed amount of adsorbed VOCs. Two approaches were used in method II to enhance the transduction properties of the composite: first, addition of DEC to the film and second, addition of PEO and DEC to the film. The two approaches and their potential effects in enhancement of cross selectivity are described.

#### 2.2.1. Addition of DEC to the Film

DEC was drop-cast on the surface of the PVDF-HFP/CB film. PVDF-HFP has a slight interaction with DEC and is, thus, considered as a gelation solvent [37]. DEC can wash away the CB clumps or interact with the small amount of oxygen groups on CB. This causes the CB to disperse with higher uniformity throughout the film. Enhancement of CB’s distribution in a composite has been reported elsewhere as a means of altering the efficacy of sensor transduction and conducting properties [20]. 

#### 2.2.2. Addition of PEO and DEC to the Film

PEO was added to the DEC and sonicated for 4 h. PEO/DEC was drop-cast on the surface of the PVDF-HFP/CB film. The use of PEO and DEC may act to transform CB particles into a PEO/CB composite. Since PEO is nonconductive, the resistivity of a PEO/CB composite would be higher than CB without PEO. This has been supported by resistance measurements across the film before and after drop-casting of PEO/DEC. Furthermore, addition of PEO to the composite alters the sensor transduction, as PEO swells differently in the presence of acetone or ethanol than PVDF-HFP does. 

### 2.3. Material Characterization 

A Nicolet iS10 FTIR spectrometer (Thermo Scientific, Waltham, MA, USA) was used to conduct FTIR experiments on films fabricated on a potassium bromide (KBr) disc (25 mm × 4 mm). Omnic software was used to estimate the FTIR peaks. An X-ray diffraction (XRD) model D8 Discover instrument with a Lynxeye XE detector (Bruker, Billerica, MA, USA) was used for further analysis of the PVDF-HFP crystal structure. A JSM-7800F field emission scanning electron microscope (JEOL USA, Peabody, MA, USA) was used to provide images and investigate the morphological properties of the PVDF-HFP/CB and pure CB on interdigitated electrodes. An EZ digital FC-7015U 100 MHz universal counter (EZ Digital, Anyang-si, Gyeonggi-do, Korea) with a 9 MHz QCM lever oscillator was used to measure frequency change of modified QCM. Acetone and ethanol concentrations were quantified using a solid phase microextraction (SPME) fiber (divinylbenzene/carboxen/polydimethylsiloxane, Supelco, Bellefonte, PA, USA) of collected samples analyzed by gas chromatography/mass spectrometry (Agilent 7890A/5975C, Santa Clara, CA, USA).

### 2.4. Sensor Experimental Set-Up

**Figure 2:**
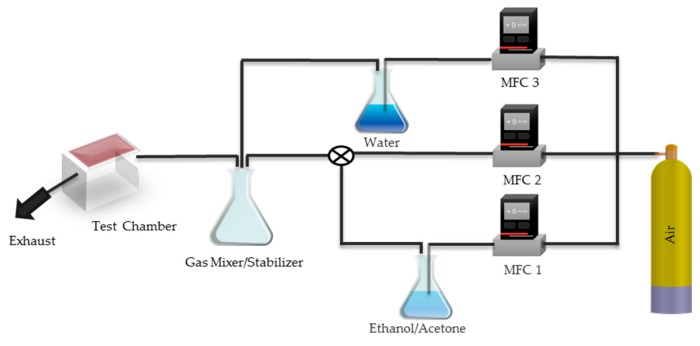
Schematic of sensor testing setup.

The experimental setup used is illustrated in Figure 2. The intended application of the sensors is for detecting VOCs in workplace environments. This is demonstrated using acetone and ethanol. The occupational safety and health administration (OSHA) endorses a humidity of 20%–60% for workplace [38]. Thus, humidified air (80% nitrogen, 19% oxygen, <1% carbon dioxide, 45% relative humidity), was used as a carrier gas to test the sensors. Purified air (Air Medipure brand, grade USP, Praxair, Danbury, CT, USA) at a fixed flow rate was bubbled through liquid acetone/ethanol in a flask. The resulting gas was diluted with pure air. Air is also bubbled through water in another flask to obtain a constant 45% RH humidity. The gasses are homogenized in the gas stabilizer. The concentration of VOCs and the humidity level is altered by controlling the flow of air (using mass flow controllers, MFC1, MFC2 and MFC3). The concentration of acetone/ethanol for given experimental conditions and flow rate were determined through gas chromatography/mass spectroscopy (GC/MS). The mixture of acetone/ethanol and air coming out of the flask was passed through a vial cooled by dry ice. The acetone/ethanol trapped inside the vial was adsorbed onto a SPME fiber and transferred to GC/MS for analysis. The samples under analysis were placed inside the test chamber and the desired measurements were taken. *R_VOC_* denotes a resistance measurement taken in the presence of ethanol or acetone, and *R_air_* represents a resistance measurement taken in the absence of ethanol/acetone. The experiments were conducted at 22 ± 1 °C. Concentrations of acetone and ethanol are reported in parts per million (ppm). 

## 3. Material Characterization Results and Discussion

The fabricated PVDF-HFP and PVDF-HFP/CB composites (with and without DEC and with and without IR treatment) were characterized using FTIR to provide information about the composite character bands, orientation of specific functional groups, polarity and the different crystalline structure of the polymer in the composite. XRD analysis was used to identify the semi-crystalline forms and scanning electron microscopy (SEM) analysis provided data about the morphology of the different PVDF-HFP/CB composites. 

### 3.1. FTIR Analysis of IR Treatment and DEC Addition

**Figure 3:**
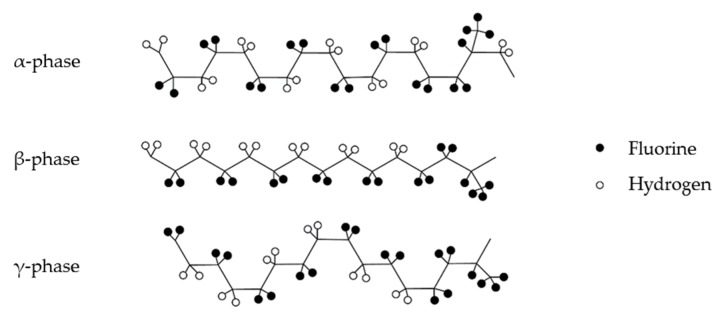
The α-phase, β-phase, γ-phase crystal structures of PVDF-HFP demonstrating the α-phase is far less polar than the β-phase. Ratio of PVDF:HFP ~ 15:1 (HFP structure on the right).

PVDF presents three primary crystalline forms. The α-phase offers a non-polar *trans*-gauche-*trans*-gauche’ conformation (TGTG’) [23]. The β-phase, which is more polar, assumes a distorted planar zig-zag. The polarity is caused by the fluorine atoms (filled) locating opposite to the hydrogen atoms (unfilled) in the crystal structure. The γ-phase assumes a polar unit cell intermediate between the helical α and zig-zag β structure [23]. PVDF with added HFP (HFP ratio of 3.5%–7.5%) also assumes these structures (Figure 3) [39]. Polymorphism of PVDF-HFP can be used to control the adsorption properties of the polymer composites. FTIR analysis was conducted for PVDF-HFP and PVDF-HFP/CB in the conditions to evaluate the effects of IR-treatment and addition of DEC to the film. Peaks reported in this section with suggested structural features noted in parenthesis (suggested features are within 1 or 2 cm^−1^ of peaks reported elsewhere for PVDF or PVDF-HFP [39,40,41,42,43,44,45,46,47,48,49,50,51,52]). 

#### 3.1.1. FTIR Analysis of PVDF-HFP and PVDF-HFP/CB

**Figure 4:**
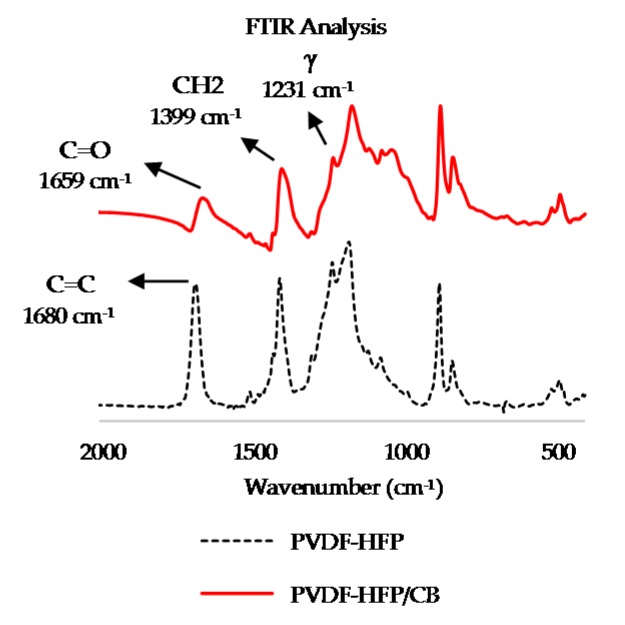
FTIR analysis of PVDF-HFP (in dotted black) and PVDF-HFP/CB (in solid red).

FTIR was undertaken for PVDF-HFP alone and PVDF-HFP/CB (Figure 4). The extensive changes to the PVDF-HFP FTIR peaks on addition of CB demonstrate the crystal structure of the polymer undergoes structural changes in formation of the composite. Peaks in PVDF-HFP at 511 cm^−1^ (CF_2_ [40], β-phase [41]), 658 cm^−1^, 838 cm^−1^ (CH_2_ rocking [33], β-phase [43]), 880 cm^−1^ (CF_2_ stretching [42], α-phase [41], 1073 cm^−1^ (out of plan deformation [44]), 1178 cm^−1^(asymmetric stretching of C-F bond [45]), 1231 cm^−1^ (γ-phase [46]), 1404 cm^−1^ (CH_2_ wagging [47]), are shifted in PVDF-HFP/CB to 509 cm^−1^, 656 cm^−1^, 835 cm^−1^ (gamma phase [46]), 876 cm^−1^, 1069 cm^−1^, 1167 cm^−1^, 1229 cm^−1^, 1399 cm^−1^. The peak at 1680 cm^−1^ (symmetrical stretching vibration of C=C [48]) has disappeared but new peaks are observed due to insertion of CB and formation of the composite. New peaks in PVDF-HFP/CB are detected at 406 cm^−1^ (α-phase [49]), 429 cm^−1^, 906 cm^−1^ (CH_2_ wag absorption in α-phase [50]), 1037 cm^−1^, 1111 cm^−1^ (C–O–C stretching [43]) and 1659 cm^−1^ (C=O stretching [51]). The C=O (1659 cm^−1^) is perceived to be attributed to oxidized CB clumps as it is not observed in PVDF-HFP.

#### 3.1.2. FTIR Analysis Post IR Treatment 

**Figure 5:**
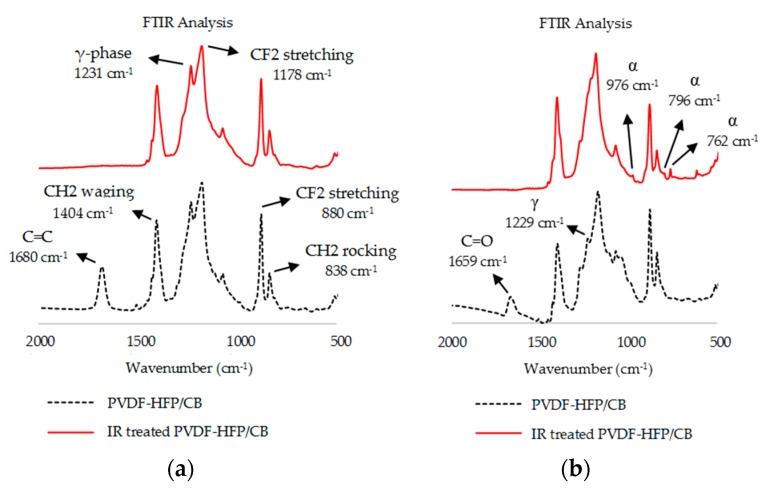
FTIR analysis of (**a**) PVDF-HFP (in dotted black) and IR treated PVDF-HFP (in solid red); (**b**) PVDF-HFP/CB (in dotted black) and IR treated PVDF-HFP/CB (in solid red) specifies formation of less polar alpha structures and loss of several bonds after IR treatment in presence of CB.

To evaluate the role of IR treatment in PVDF-HFP/CB composite, FTIR analyses were conducted on both PVDF-HFP and PVDF-HFP/CB before and after IR treatment. The FTIR peak of PVDF-HFP at 1680 cm^−1^ (C=C) disappears after IR treatment but the FTIR peaks corresponding to α, β and γ crystal structures of IR-treated PVDF-HFP were unchanged from FTIR peaks in untreated PVDF-HFP (Figure 5a). On the other hand, FTIR signal of the PVDF-HFP/CB composite did shift quite significantly after IR treatment (Figure 5b).

IR treatment caused the intensity of FTIR peaks to be decreased at 1037 cm^−1^, 1111 cm^−1^ (C–O–C stretching [47]), 1229 cm^−1^, 1504 cm^−1^ (CH_2_ bond [52]), 1659 cm^−1^. The new FTIR peaks at 762 cm^−1^ (α-phase [53]), 796 cm^−1^ (α-phase [53]), 976 cm^−1^ (α-phase [53]) after IR treatment support the realization of more α-phase structure in the composite. Spin casting of thin films elutes the PVDF-HFP polymer in a mixture of the more polar β or γ crystalline structure along with some α structure under our experimental conditions (FTIR peaks in Section 3.1.1). IR treatment transforms the film so that it has more α structures in the composite. Changing the polymer from the more-polar β or γ crystalline structure to a non-polar α changes the composite’s adsorption properties. This is further discussed in Section 4.1.2. It was initially unexpected to the researchers that IR treatment of PVDF-HFP/CB induced a change in crystalline structure, while the same treatment of PVDF-HFP did not. However, it has been shown that heating PVDF-HFP up to melting point and cooling it slowly induces formation of α-phase structures. It is believed that the CB particles in the composite absorb IR energy and locally heat the PVDF-HFP in the composite. Slow cooling of the CB-heated PVDF-HFP then causes the formation of α-phase structures seen by FTIR. 

#### 3.1.3. FTIR Analysis Post Addition of DEC 

**Figure 6:**
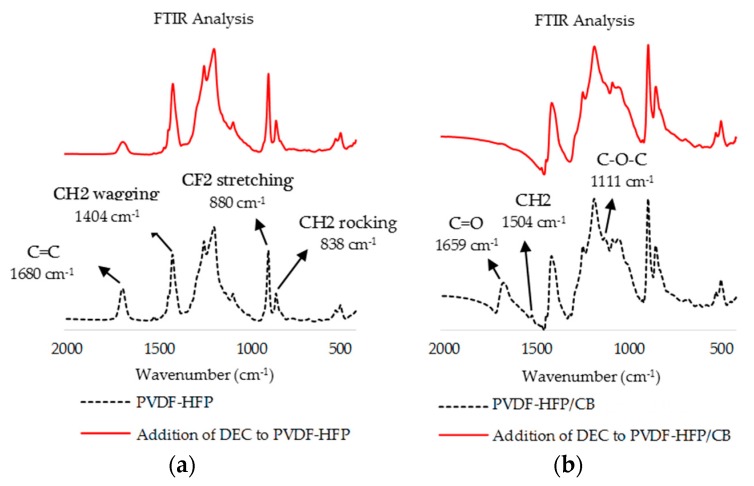
FTIR analysis of (**a**) PVDF-HFP (in dotted black) and PVDF-HFP after addition of DEC (in solid red); (**b**) PVDF-HFP/CB (in black) and PVDF-HFP/CB after addition of DEC (in solid red).

FTIR analysis were conducted to further investigate the role of addition of DEC to PVDF-HFP and to PVDF-HFP/CB. The results are presented in Figure 6. FTIR analysis (Figure 6a) indicates that the crystalline order of PVDF-HFP is not considerably affected when DEC is added to the PVDF-HFP alone. The peaks at 483 cm^−1^, 511 cm^−1^, 601 cm^−1^, 658 cm^−1^, 838 cm^−1^, 880 cm^−1^, 1073 cm^−1^, 1178 cm^−1^, 1405 cm^−1^, 1429 cm^−1^ do not change after addition of DEC to the polymer. Not only the frequencies but also the intensities remain similar. The polymer also remains amorphous, as the peaks at 838 cm^−1^ and 880 cm^−1^ (associated with amorphous structure of the film) stay unchanged [2]. FTIR spectra for PVDF-HFP/CB and PVDF-HFP/CB after addition of DEC demonstrates noteworthy changes (Figure 6b). The disappearance of the peaks at 1111 cm^−1^ (C–O–C stretching [47]), 1504 cm^−1^ (CH_2_ bond [52]), 1659 cm^−1^ (C=O bond [51]) and decrease in intensity of 1299 cm^−1^ (mixture of CF_2_ stretching, CC stretching and CH_2_ rocking [54]) are detected after addition of DEC to the PVDF-HFP/CB. The most striking difference, however, is that the carbonyl bond (C=O 1659 cm^−1^) in PVDF-HFP/CB disappears after addition of DEC to the composite. The loss of the peak at 1659 cm^−1^ indicates that oxidized CB clumps are either washed away or reduced by the DEC. In either event, addition of DEC creates a more uniform film across the composite. This is believed to enhance the transduction property, which is analyzed in Section 4.2.

### 3.2. XRD Analysis

**Figure 7:**
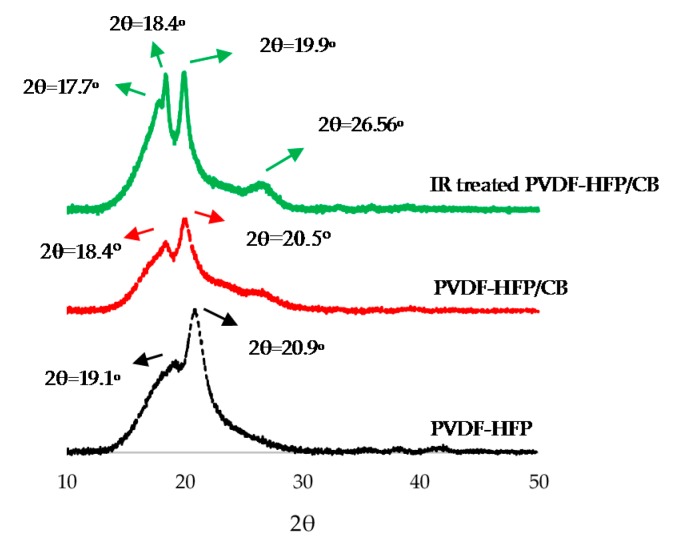
XRD of PVDF-HFP (in black), PVDF-HFP/CB (in red) and IR treated PVDF-HFP/CB (in green) (2θ between 10° to 50°).

The XRD patterns for PVDF-HFP, PVDF-HFP/CB and IR treated PVDF-HFP/CB are illustrated in Figure 7. The data were collected from 2θ = 10° to 50° at a rate of 1°·s^−1^. This analysis illustrates the semi-crystalline property of the PVDF-HFP. PVDF-HFP peaks are located at 19.1° and 20.9°. PVDF-HFP/CB shows peaks at 18.4° and 20.05°. XRD analysis also shows changes in crystal structure of PVDF-HFP/CB due to IR treatment. The peak at 20.05° for PVDF-HFP/CB shifts to 19.9° after IR treatment and new peaks at 17.7° and 26.56° are observed. This confirms the formation of α phase as the peaks at 17.6°, 18.4° and 26.56° represent α phase [23]. IR treated PVDF-HFP did not show marked differences than untreated PVDF-HFP (data not shown).

### 3.3. SEM Analysis

**Figure 8:**
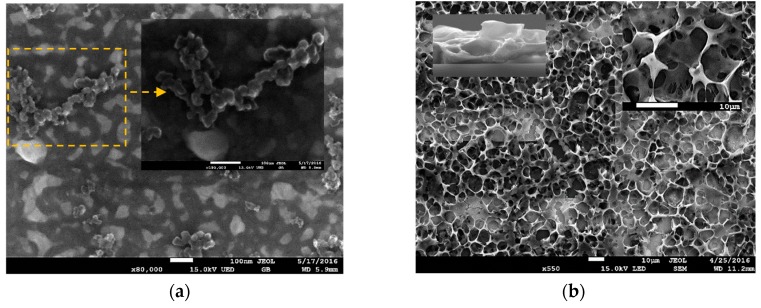
(**a**) SEM images of CB (black pearl 2000). A CB structure at higher magnitude is shown in the inset. Scale bar 100 nm; (**b**) SEM image of thin PVDF-HFP/CB film on the IDE. Note the porous structure. The film cross section is shown in the inset top left. The pores at higher magnification are shown in the inset top right. Scale bar 10 μm.

SEM images of CB (black pearl 2000) and the PVDF-HFP/CB porous films are presented (Figure 8.). The CB structures is shown in Figure 8a. The CB particle size was measured and estimated to be 36 nm. The image of a PVDF-HFP/CB composite on top of the IDE is shown in the Figure 8b. The morphology and the porous thin film structure is demonstrated. The high surface area and the micron-size pores of the thin film are illustrated in the image. This indicate the available large surface for interaction between the VOCs and the film. In addition, the micron-size pores provide space for addition of a thin polymer such as PEO to the composite to enhance the adsorption ability of the composite. Cross section image of the film and the pores at higher magnification are shown in the insets. The average film thickness was estimated to be 4.2 µm.

## 4. Sensor Results and Discussion

Sensors fabricated with the methods and materials described in Section 2 were tested using the setup discussed in Section 2.4. The results from the sensors are presented and discussed in this section. For the experiments, a set of three devices were fabricated at the same batch and tested simultaneously. Results are presented in terms of relative response (*RR*) according to the following equation:

(1) *RR_VOC_* = *(R_VOC_* − *R*_air_*)/R*_air_ × 100

The presented results show the average response of the three devices with the standard deviations as error bars. Devices with and without enhancement were tested with acetone and ethanol at the same time. Sensor selectivity (*SS*) is defined as *RR*_ethanol_ divided by *RR*_acetone_ (using 2300 ppm concentration via linear regression analysis; results obtained at different concentrations are similar):

(2) *SS* = *RR*_ethanol_/*RR*_acetone_

The sensing mechanism of PVDF-HFP/CB composite can be described based on percolation theory in the matrix [20]. CB particles form conducting paths in the PVDF-HFP matrix. Swelling of PVDF-HFP in response to VOCs increases the separation between CB structures and increases the sensor resistance. The transduction efficiency of the composite depends on the swelling properties of the polymer that separate two CB structures and also the distribution and uniformity of the CB structures across the film. Methods to alter the adsorption ability and transduction efficiency of PVDF-HFP/CB are described in Section 2. In similar studies of sensors comprised of polymer with conductive filler (including CB) [2,55,56], or modeling this type of sensor [19,20], the polymer has been shown to play the major role. 

**Figure 9:**
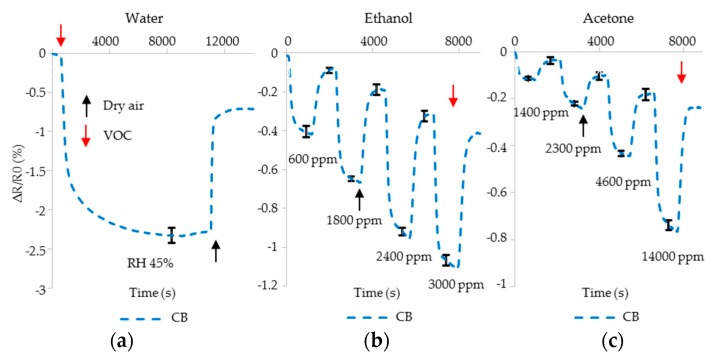
Resistance change of the CB sensors as they are exposed to (**a**) water vapor with RH 45%; various concentration of (**b**) ethanol and (**c**) acetone.

The sensors reported on in Section 4.1 and following are a mixture of polymer and CB. To better understand the contribution of CB, sensors of CB alone were fabricated and tested using VOCs and dry air. A sensor was built by drop-casting CB (without PVDF-HFP) onto an IDE and exposing it in turn to a VOC followed by dry air. The results are shown in Figure 9. CB resistivity decreases due to exposure to water vapor, ethanol and acetone and does not fully return to pre-exposure levels. For water vapor, equilibrium is reached after 2 h (7200 s). For ethanol and acetone, the long-term effect is also present. Sensors with CB incorporated into a PVDF-HFP polymer may exhibit a similar negative drift when exposed to ethanol or acetone. On the other hand, swelling of PVDF-HFP when exposed to ethanol or acetone causes an increase in resistance. Effects of incomplete desorption of VOCs from PVDF-HFP would likely cause a positive baseline drift.

### 4.1. Method I

Adsorption properties of the film are altered by addition of a thin PEO layer on the composite or through IR treatment of the film. In both cases the ability of the composite to adsorb acetone or ethanol are altered.

#### 4.1.1. Addition of a PEO Layer on the Film

**Figure 10:**
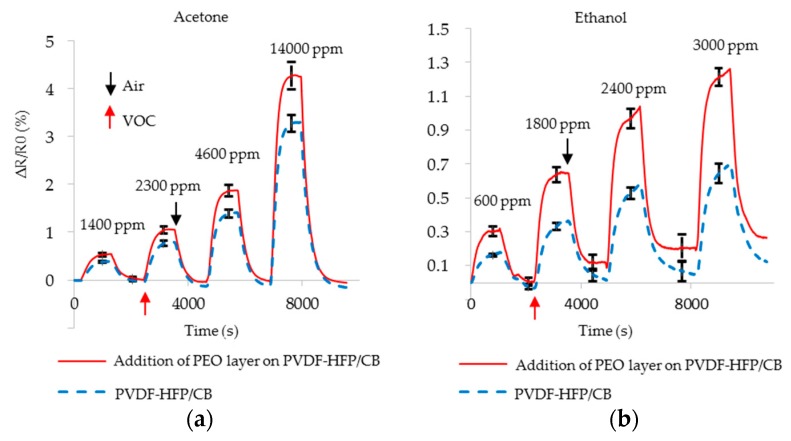
Resistance change of the PVDF-HFP/CB sensors with PEO layer (red solid) and without PEO layer (blue dotted) as they are exposed to various concentration of (**a**) acetone; (**b**) ethanol.

The PEO, dissolved in water, was cast onto the film as described in the Methods section. The addition of PEO layer on the film alters the adsorption of both ethanol and acetone to the composite. QCM analysis was conducted to confirm and estimate the mass of PEO. There is a linear relation between the frequency of QCM and the mass of the material for thin films [55]. QCM frequency was measured before and after addition of PEO layer. Addition of PEO layer on the PVDF-HFP/CB dropped the frequency by 1003 Hz, corresponding to 3.4% mass of PVDF-HFP. The film was analyzed by XRD analysis both before and after addition of PEO, with no change in crystal structure observed. Resistance studies before and after addition of PEO layer to the PVDF-HFP/CB show that addition of PEO layer does not alter the sensor’s resistance (percent change in resistance <1.0%). XRD and resistance studies both confirm that PEO does not incorporate into the composite film, but likely attaches to the surface as a thin layer, which is supported by QCM analysis. This is likely because PEO has a tendency to attach to hydrophobic materials such as PVDF-HFP [57]. It can also attach to the surface of CB. PEO has a low glass transition temperature (−67 °C) which offers a fast response to a VOC. This thin layer of PEO on the surface of PVDF-HFP/CB is used to enhance the adsorption of VOCs. The response of the sensors with and without the thin PEO layer are studied for different concentrations of acetone and ethanol (Figure 10). Addition of PEO layer results in enhancement of the sensor’s relative response to both acetone and ethanol. The enhancement relative to untreated composite varies from 62% to 68% for various concentration of ethanol. The enhancement is lower for acetone than ethanol (22% to 30%). The *SS* for the untreated sensors is 0.63 but increases to 0.83 after addition of PEO layer on PVDF-HFP/CB (*SS*_ORIG_ = 0.63; *SS*_PEO_ = 0.83). The significant change in cross-selective enhancement is a powerful tool for building VOC-selective sensors. It is noted that the sensors exposed to ethanol experience slight increased positive baseline drift after addition of PEO. According to the sensing mechanism described above, VOCs trapped in PVDF-HFP and not fully released would cause the sensor resistance to exhibit a positive baseline drift. PEO interacts with ethanol to form a unique crystalline structure [58]. This likely causes incomplete ethanol desorption and thus enhanced positive drift after addition of PEO.

#### 4.1.2. IR Treatment

**Figure 11:**
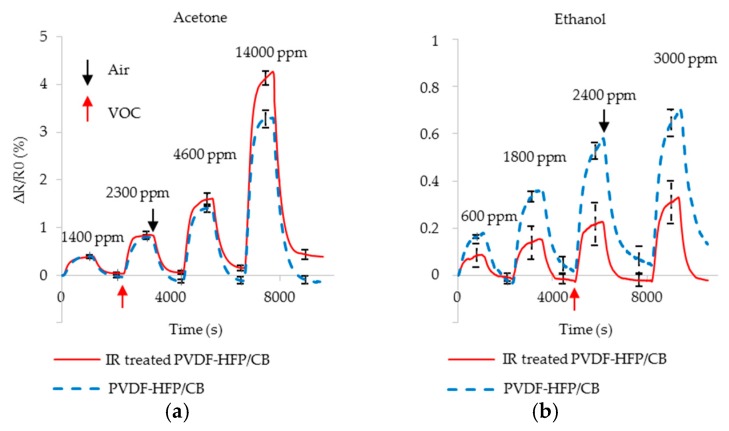
Resistance change of the IR treated (red solid) and non-treated (blue dotted) PVDF-HFP/CB sensors due to the exposure to various concentration of (**a**) acetone; (**b**) ethanol.

IR treatment increases the amount of α structures in PVDF-HFP/CB as shown in Section 3.1.2. This decreases the polarity of the composite. The decrease in polarity of the film reduces its strength in adsorbing more polar VOCs. Here, the composite’s capacity to detect more polar ethanol is decreased. The sensors are exposed to the various concentrations of acetone and ethanol in 45% RH humid air, and the results are shown in Figure 11.

The maximum resistance change in each cycle for acetone is similar for both before and after IR treatment. However, the sensor’s relative responses is decreased up to 57% for ethanol. In addition, the slight positive drift observed before IR treatment is replaced with a slight negative drift, likely due to CB/ethanol interactions similar to those observed in Figure 9b. This treatment provides a second and opposite method to enhance cross-selectivity. IR treated film adsorbs less ethanol than untreated film. This causes the IR treated film to swell less than untreated film which results in a smaller resistance change. As the result, *SS_IR_* decreases to 0.32.

### 4.2. Method II

DEC is added to the films in order to redistribute CB more uniformly throughout the PVDF-HFP/CB composite, thereby seeking to increase transduction efficiency. FTIR analysis of PVDF-HFP/CB after addition of DEC suggested a decrease in CB clusters (Section 3.1.3). As PEO enhances cross-selectivity, PEO/DEC was also added to the films for an enhanced cross-selective response. Resistance studies before and after addition of DEC demonstrated that addition of DEC to the film decreases the initial resistance of the sensors up to 61%. The decrease in carbon clustering and decrease in initial resistance suggest the formation of a more uniform composite. Breaking down bigger clusters of CB to smaller structures increases the average contact of PVDF-HFP and CB. This enhances the transduction efficiency and sensor sensitivity to both acetone and ethanol.

**Figure 12:**
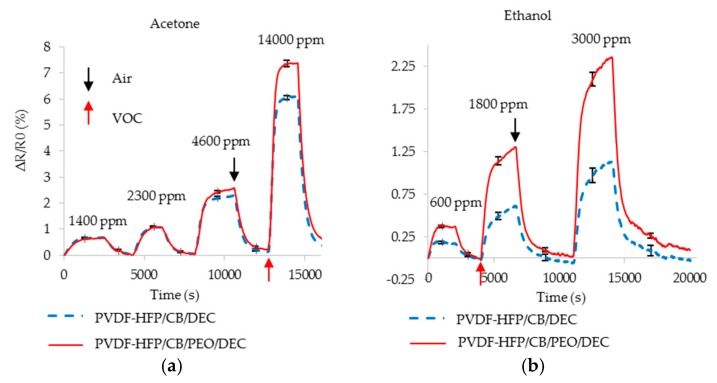
Resistance change of PVDF-HFP/CB film after addition of DEC (dotted blue) is compared with addition of PEO/DEC (solid red) at various concentration of (**a**) acetone; (**b**) ethanol.

To show the enhancement the relative response of PVDF-HFB/CB after addition of PEO/DEC to the composite is compared to the system after addition of DEC alone (Figure 12). As expected, upon addition of DEC alone, both films offer a higher sensor response to acetone (up to 73% better than original PVDF-HFP/CB film) and ethanol (up to 74%). Addition of PEO/DEC to the film further enhances the transduction efficiency and sensor’s sensitivity to ethanol (up to 197% over PVDF-HFP/CB) while displaying only a modest further enhancement to acetone (up to 89%). These modifications lead to *SS*_DEC_ = 0.89 and *SS*_PEO/DEC_ = 1.74. Thus, the modifications to both adsorption capability (addition of PEO alone, *SS*_PEO_ = 0.83) and improvement in transduction efficiency (addition of DEC alone, *SS*_DEC_ = 0.89) appear to be cumulative (addition of PEO and DEC, *SS*_PEO/DEC_ = 1.74). This shows addition of PEO/DEC to the film not only increases the sensitivity but also improves the sensors’ cross-selectivity when used with addition of DEC to the film. The higher change in resistance also suggests that the use of PEO and DEC potentially transforms CB particles into a PEO/CB composite. Swelling of PEO increases the separation between CB particles which increases the resistivity of PEO/CB composites. This increases the sensitivity of the film and also enhances the cross-selectivity of system.

### 4.3. Sensor Degradation 

To study the degradation of the composite materials, five sensors of PVDF-HFP/CB were fabricated and tested over a 25 day periodically. The sensors were stored in ambient air. The sensors were tested for their response using synthetic air with humidity of 45% RH. The standard deviation (σ) of the sensors’ resistance before and after exposure was less than 1.1% of initial resistance, indicating a good stability of the PVDF-HFP/CB composite material. No decreasing or increasing pattern in the sensor’s initial resistance and sensors’ response was observed over this period. Acetone was the VOC used for these tests, but a similarly robust result would be expected from ethanol or any other VOC. The excellent stability is believed to be due to hydrophobic properties of PVDF-HFP that prevent moisture absorption, and the high stability of the materials against oxygen, nitrogen, and carbon dioxide. 

## 5. Conclusions

Two methods for cross-selectivity enhancement of porous thin PVDF-HFP/CB film based sensors for acetone and ethanol have been presented and discussed. The first method, method I, uses techniques to alter the adsorption properties of the sensor film. These techniques included addition of a layer of PEO to improve the adsorptions of ethanol and IR treatment of the film to reduce the sensitivity to ethanol. It is interesting that IR treatment of PVDF-HFP had no effect on the crystal structure of the PVDF-HFP, while IR treatment of PVDF-HFP/CB did cause a change in the crystal structure of the PVDF-HFP. It is suggested that the IR treatment caused localized heating of the CB particles, which subsequently cooled over time. Slow cooling of heated PVDF-HFP (5%) has been shown to favor formation of alpha phase [39]. This resulted in an enhanced cross-selectivity of ethanol and acetone. Addition of PEO layer increased the sensors relative response by up to 30% for acetone and 68% for ethanol. In terms of selectivity, this led to *SS*_ORIG_ = 0.63 and *SS*_PEO_ = 0.83. IR treatment did not significantly affect acetone response, and it reduced the ethanol relative response by up to 57% leading to *SS*_IR_ = 0.32. The second method, method II, uses techniques to improve the electrical transduction efficiency of the sensor film. Addition of DEC or PEO/DEC enhanced the transduction properties across the board but also, for PEO/DEC, significantly increased selectivity; *SS*_DEC_ = 0.89 and *SS*_PEO/DEC_ = 1.74. These modifications altered sensor selectivity from 0.32 to 1.74. The fabrication methods and characterization results for the sensors have been presented and discussed. The sensors were tested with various concentrations of ethanol and acetone in 45% RH humid air at 22 °C, thereby mimicking typical environmental conditions. The presented methods can be utilized in a single film with multiple IDEs to realize a cross-selective sensor array for ethanol and acetone in the presence of high humidity applications.

## Figures and Tables

**Table 1:** Reported percentage of resistance change in response to ethanol and acetone for resistor based sensors at room temperature in different studies.

Material	Acetone	Ethanol
Poly(4-vinylphenol)/CB	4.2% (49,000 ppm) [24]	2.3% (10,000 ppm) [24]
PMMA/f-CNT	30% (55,000 ppm) * [25]	1.7% (5000 ppm) * [26]
Iso-butyl methyl ketone/CNT	Not available	1.25% (1000 ppm) [27]
PDLC/CNT	4% (1800 ppm) * [28]	Not available
Network of CNT	1% (3200 ppm) * [29]	11.3% (77,000 ppm) * [30]
Network of oxidized CNT	3.3% (3200 ppm) * [29]	4.2% (5000 ppm) * [31]
Organic thiol capped GNP	0.5% (500 ppm) * [13]	1.15% (1500 ppm) [32]

* Indicates results are estimated from the figures or data in the study.
